# Prelacteal feeding and associated factors among newborns in rural Sidama, south Ethiopia: a community based cross-sectional survey

**DOI:** 10.1186/s13006-018-0149-x

**Published:** 2018-02-20

**Authors:** Nana Chea, Anteneh Asefa

**Affiliations:** 1 School of Public and Environmental Health, College of Medicine and Health Sciences, Hawassa University, Hawassa, Ethiopia; 2 Nossal Institute for Global Health, The University of Melbourne, Melbourne, Australia

**Keywords:** Prelacteal, Feeding, Breastfeeding, Newborns, Mothers

## Abstract

**Background:**

The practice of giving prelacteal feeds deprive a newborn of valuable nutrients and expose the newborn to risks of infection. Despite its negative health outcomes, prelacteal feeding prevails in Ethiopia. Therefore, the current study was undertaken to assess the prevalence of prelacteal feeding practices and its associated factors in a rural community in south Ethiopia.

**Methods:**

We conducted a community based cross-sectional study of 597 mothers of children aged less than six months. Mothers were selected using a multistage cluster sampling technique from Hawela Tula, a rural catchment under Hawassa City Administration. Newborns exposed to any foods, substances or drinks other than human milk before the initiation of breastfeeding or during the first three days of birth were regarded as receiving prelacteal feeds. Descriptive summaries were done to present the main findings; bivariate and multivariate logistic regression analyses were undertaken to identify variables associated with prelacteal feeding practices.

**Results:**

Among the total infants, 25.5% (95% confidence interval [CI] 23.5%, 27.5%) were found to be exposed to prelacteal feeds. Boiled water (36.8%) and fresh butter (32.2%) were the top two prelacteal foods. The prevalence of prelacteal feeding was higher among infants whose mothers are housewives, and among infants born to mothers aged between 21 and 34 years. Almost two-third (64.3%) of mothers who exposed their newborn to prelacteal feeds did so with advice from their parents. Mothers who had poor knowledge on breastfeeding were nine times more likely to practice prelacteal feeding compared to those with good knowledge (adjusted odds ratio [AOR] 8.9, 95% CI 4.2, 18.7). Lack of knowledge on the risks associated with prelacteal feeding (AOR 6.8; 95% CI 2.6, 17.8) and misconceptions about breastfeeding (AOR 8.1; 95% CI 3.9, 16.6) were associated with prelacteal feeding. However, mothers’ place of delivery and attendance at breastfeeding counseling sessions showed no association with the practice of prelacteal feeding.

**Conclusions:**

Prelacteal feeding is commonly practiced in the study area. Raising women’s awareness on the consequences of prelacteal feeding is warranted. Involving parents of women when promoting optimal infant feeding practices should be emphasized.

## Background

There is an evidence that improving breastfeeding practices, that includes avoiding prelacteal feeding, would help to save the lives of around 823,000 children under five years of age, annually [1]. In particular, 45% of neonatal infectious deaths, 30% of diarrheal deaths, and 18% of acute respiratory deaths among children aged less than five years are associated with prelacteal feeds [2]. In addition to this, the risk of mortality among newborns aged 2 to 28 days is reported to be higher among prelacteal fed infants, especially low birth weight infants [3]. Prelacteal feeds are foods or substances or drinks other than human milk that are given to newborns before breastfeeding initiation or before breast milk comes in, usually in the first few days of life. However, there are varying definitions and terminologies used to describe such a feeding practice. In this study, we opted to use the term prelacteal feeding in order to align with the working definitions of the Demographic and Health Surveys used in various countries. Honey, butter, goat’s milk, cow’s milk, boiled water, and clean water are the common prelacteal foods usually given to newborns [4–7]. Prelacteal feeding deprives an infant the nutrients that it needs in the first six months of life, that would be obtained by exclusive breastfeeding [7]. Furthermore, prelacteal feeds predispose newborns to pathogenic contaminants [8, 9], create physiological disruptions in the immature gastrointestinal, and renal systems [8], and discourage newborns from initiating breastfeeding [6].

There is a widespread practice of prelacteal feeding in developing countries including Ethiopia. According to the Ethiopian Demographic and Health Survey reports, prelacteal feeding practice showed modest reduction between 2005 (29%) and 2011 (27.1%) [10, 11]. The level in the multi-ethnic Southern Nations, Nationalities and Peoples Region (SNNPR) was found to be 10% in 2011, although variation exists among administrative zones. Level of prelacteal feeding practice in Boricha district, a district geographically closer to the current study site, was 40.6% in 2011 [12].

Belief that prelacteal feeds serve as laxatives or hydrating agents is among the main reasons for practicing prelacteal feeding by Ethiopian communities [13, 14]. In addition, there are communities that believe prelacteal feeds help in clearing meconium and thereby prevent the risk of regurgitation and aspiration. However, it is believed that there are still factors driving the widespread practice of prelacteal feeding that are not yet well understood [15].

One part of the core strategic agenda of the Ethiopian Health Sector Transformation Plan (2015/16–2019/20) is reducing neonatal and child morbidity and mortality [16]. In light of this, the country is implementing the national infant and young child feeding (IYCF) guideline to promote optimal IYCF practices and discourage prelacteal feeding, in accordance with the recommendations of the United Nations Children’s Fund (UNICEF) and the World Health Organization (WHO) [17–19].

This study assessed the level of prelacteal feeding practices and associated factors among newborns aged less than six months in Hawela Tula, southern Ethiopia. As there are diverse cultural practices among Ethiopian communities, evidence generated by this study will assist in developing context specific interventions to halt prelacteal feeding practice and promote optimal IYCF practice.

## Methods

### Study design and setting

Hawela Tula is a rural catchment that is under the scope of Hawassa City Administration, which has another seven urban sub-cities. Hawela Tula has 1 urban and 11 rural kebeles (the smallest administrative unit in Ethiopia), and an estimated total population of 163,872, from which nearly 48% are females. There are six functional health centers and 17 functional health posts which provide primary healthcare services to the catchment population. Health education on infant feeding is among the services rendered at community level by health extension workers. The common staple food in the study area is false banana, which is called “Wese” in Sidama Afoo, the local language predominantly used in the study site. A cross-sectional study design that deployed quantitative interviews of mothers of children was conducted to estimate the prevalence of prelacteal feeding.

### Study participants and eligibility criteria

Biological mothers who had a child or children aged less than six months in Hawela Tula were the source population of the study. Biological mothers who did not live for six consecutive months in the study area, who had communication problems (unable to speak or hear), and who had serious health problems (critically ill mothers) were excluded from the study.

### Sample size and sampling

A single population proportion formula was used to estimate the sample size of this study with the assumptions of: 5% precision, 95% confidence, 10% non-response rate, and a design effect of 1.5. A 38.8% estimated prevalence of prelacteal feeding practice was considered from a previous study conducted in Raya Kobo district (rural based study), north eastern Ethiopia [5]. Accordingly, the final calculated sample size was 597. Proportionate allocation of samples to kebeles (based on the number of target respondents in each kebele) was made in a multistage fashion; initially four kebeles were randomly selected from the 11 rural kebeles. Following, initial numbering of all households with target respondents in the four kebeles was conducted to generate a sampling frame for the selection of households using systematic random sampling approach. In scenarios where there were more than one potential respondent in a household, simple random sampling was done to select one. Accordingly, 154, 190, 157 and 149 mothers were allocated to Fincawa, Tula Rural, Tullo, and Gamato Gale kebeles, respectively.

### Data collection, processing and analysis

Structured and pretested questionnaire initially prepared in English and later translated to the local language widely used in the study area (Sidamu Afoo) was used to collect data. The questionnaire was adopted from the Ethiopian Nutritional Survey Tools and the Ethiopian Demographic and Health Survey [11, 20]. Data was collected by trained data collectors who are health professionals and experts in the local language. Face-to-face interview technique was conducted at the study participants’ house; revisiting was scheduled for unavailable or busy potential respondents.

To assess the outcome variable (prelacteal feeding), a single question which was narrated as “did you give any food/fluid to your newborn before the initiation of breastfeeding or in the first three days of birth?” was forwarded to mothers; a dichotomized possible response of “Yes” or “No” were offered to respondents. Therefore, newborns whose mothers responded “Yes” to the aforementioned question were regarded as prelacteal fed. Additionally, knowledge and misconception on breastfeeding and health services utilization history of mothers were assessed using closed-ended questions. Five questions (the first milk of the breast is not important to a newborn, giving fluids/liquids prior to initiating breastfeeding is important to the health of a newborn, a breastfed newborn will get hungry if not given additional food within 24 h of birth, a newborn will get thrush if its mouth is not cleaned with water after breastfeeding, and women with small breasts have difficulty producing enough breastmilk) with possible responses of “agree” or “disagree” were used to assess respondents’ misconception on breastfeeding. Respondents who responded “agree” to at least one of the five questions were considered to have misconception.

Data were entered into and analyzed using SPSS for windows version 20 software. Descriptive statistics like percentages and frequency distributions were displayed using tables and figures. Binary logistic regression analysis was performed to assess the association between the various explanatory variables and the outcome variable. Age of child, mother’s religion, mother’s age, mother’s occupation, mother’s level of education, mother’s number of children, antenatal visit, place of delivery, knowledge on the risks of prelacteal feeding, receipt of counseling service on breastfeeding, knowledge on breastfeeding, misconception on breastfeeding, and cultural belief on breastfeeding were included in the bivariate analysis. Additionally, multivariate logistic regression was performed to control for the effects of potential confounders. Variables with *p -*value < 0.25 in the binary logistic regression analysis were included in the multivariate logistic regression analysis. The Hosmer-Lemeshow goodness-of-fit was used to test for model fitness. Adjusted odds ratio with 95% confidence interval was reported. A *p* - value of less than 0.05 was used to declare presence of statistical significance.

### Data quality assurance

To assure data quality, the following measures were undertaken: data collectors and supervisors were trained for two days on the objectives, relevance of the study, confidentiality of information, respondent’s right, importance of pretest, principles of informed consent, and techniques of interviewing. Furthermore, adjustments were made to the data collection tool after pretesting it in Alamura Kebele (one of the 11 kebeles not selected for the actual study) taking 5% of the sample size. Depending on the feedback generated from the pretest, modifications were made to improve the clarity, understandability, and simplicity of the messages embedded in the questionnaire. Completed questionnaires were checked for completeness and accuracy on a daily basis during the entire data collection.

### Operational definition of terms

Prelacteal feeds: Foods, substances, or drinks other than human milk that are given to newborns before breastfeeding initiation or before breast milk comes in, usually on the first few days of life.

Prelacteal feeding: The administration of any foods or liquids other than breast milk to an infant during the first three days after birth.

Index child: The last baby of a mother, less than six months of age, about whom feeding practice is being assessed.

Knowledge on breastfeeding: mothers’ knowledge was categorized into three as follows

- Good knowledge on breastfeeding: provision of correct answers to all (three) breastfeeding knowledge assessment questions
- Moderate knowledge on breastfeeding: provision of correct answers to two of breastfeeding knowledge assessment questions
- Poor knowledge on breastfeeding: provision of correct answers to one or none of breastfeeding knowledge assessment questions

## Results

### Sociodemographic characteristics

A total of 597 mothers have agreed to participate in the study, making the response rate 100%; revisiting was made for unavailable and busy participants at initial contact. The mean ± standard deviation (SD) age of the respondents was 26.2 ± 5.2 years (Table 1). Among the participants, 76.9% were Christian Protestant in religion. About one-third (39.0%) of participants did not attend any formal education. Nearly four-fifths (79.2%) of respondents were housewives and 44.1% had two children or fewer.

**Table 1:** Sociodemographic characteristics of respondents, Hawela Tula, SNNPR, 2016

Variables	Frequency (%)
Age (in completed years)	
≤ 20	78 (13.1)
21–34	472 (79.1)
≥ 35	47 (7.9)
Mean ± SD	26.2 ± 5.2
Educational status	
No formal education	233 (39.0)
Formal education (grade one and above)	364 (61.0)
Religion	
Protestant	459 (76.9)
Catholic	55 (9.2)
Muslim	78 (13.1)
Others	5 (0.8)
Occupation	
Housewife	473 (79.2)
Merchant	105 (17.6)
Government Employee	11 (1.8)
Private employee	5 (0.8)
Others	3 (0.5)
Number of children	
≤ 2	263 (44.1)
3–4	214 (35.8)
≥ 5	120 (20.1)

### Knowledge on breastfeeding

Respondents were asked three questions to assess their knowledge on breastfeeding (Table 2). Accordingly, 37% of mothers had good knowledge on breastfeeding. More than half (62.1%) of the respondents knew that breastfeeding should begin within one hour after birth; 72.5% of respondents knew that feeding fluids/substances before the initiation of breastfeeding pose a risk such as diarrhea and vomiting. Additionally, 80.6% of the respondents knew that newborns should be fed colostrum.

**Table 2:** Respondents’ awareness, knowledge, and misconception on breastfeeding, Hawela Tula, SNNPR, 2016

Variables	Frequency (*n*/%)
How soon after birth should a newborn start breastfeeding?	
Immediately or less than an hour	371 (62.1)
One hour after delivery	214 (35.8)
I don’t know	12 (2.0)
What should a mother do with the ‘first milk’ or colostrum?	
Feed the newborn	481 (80.6)
Discard it	116 (19.4)
Do you know the risks associated with giving fluids/substances other than breast milk before initiation of breastfeeding?	
Yes	433 (72.5)
The commonest risk a mother knows (*n* = 433)	
Diarrhea	133 (22.3)
Vomiting	253 (42.4)
Other diseases	28 (4.7)
Not sure	19 (3.2)
No	164 (27.5)
Maternal knowledge on breastfeeding	
Poor knowledge	197 (33%)
Moderate knowledge	179 (30%)
Good knowledge	221 (37%)
Misconception on breastfeeding (*n* = 597) – Agree	
The first milk of the breast is not important to a newborn	134 (22.4)
Giving fluids/liquids prior to initiating breastfeeding is important to the health of a newborn	99 (16.6)
A breastfed newborn will get hungry if not given additional food within 24 h of birth	271 (45.4)
A newborn will get thrush if its mouth is not cleaned with water after breastfeeding	137 (22.9)
Women with small breasts have difficulty producing enough breastmilk	30 (5.0)

### Belief and misconception on breastfeeding

In this study, only 64 (10.7%) of the particpants reported that giving other fluids/foods to newborns in the first three days is a social norm in their community. However, among the remaining 533 respondents who reported non-existence of any social norm to give fluids/foods to newborns, it was claimed that prelacteal feeding is practiced due to personal advice from grandparents (64.3%), the belief that prelacteal feeding serves as a cleansing agent (24.1%), and inadequate breast milk production (11.6%). Besides, study participants’ misconception on breastfeeding was assessed using five questions. Accordingly, 45.4% of respondents believed that their baby will be hungry if not given other fluids in addition to breast milk within 24 h after birth. On the other hand, 22.4% of respondents reported that colostrum is not important for a newborn (Table 2).

### Healthcare service utilization

Almost all (94.5%) of respondents have attended at least one antenatal visit during the pregnancy of their index child, of which only 24.5% had four antenatal visits (Table 3). Among mothers who attended an antenatal visit, 84.9% reported to have received counseling on breastfeeding. Regarding the place of delivery, 33.8% of respondents gave birth to their index child at home, while the remaining 66.2% delivered at a health facility. Furthermore, 86.4% of the respondents claimed to have ever received health information on breastfeeding/infant feeding, mostly from health extension workers.

**Table 3:** Maternal health services utilization among study participants, Hawela Tula, SNNPR, 2016

Variables	Frequency (%)
Received antenatal care during index pregnancy	
Yes	564 (94.5)
No	33 (5.5)
Number of antenatal visits during index pregnancy (*n* = 564)	
One	21 (3.5)
Two	94 (15.7)
Three	303 (50.8)
Four	146 (24.5)
Received counseling on breastfeeding at antenatal care visit (*n* = 564)	
Yes	507 (84.9)
No	57 (9.4)
Place of delivery of index child	
Home	202 (33.8)
Health facility	395 (66.2)
Ever received any information on breastfeeding/infant feeding	
Yes	516 (86.4)
No	81 (13.6)
Source of information on breastfeeding/infant feeding (*n* = 516)	
Health facility	415 (69.5)
Traditional birth attendant	16 (2.7)
Family/friends/relatives	15 (2.5)
One to five mothers’ networking	70 (11.7)

### Prevalence of prelacteal feeding

Among the study participants, 25.5% (95% CI 23.5%, 27.5%) reported that they have given prelacteal feeds to their newborn in the first three days of birth (Table 4). Among respondents who practiced prelacteal feeding, 36.8% and 32.2% gave boiled water and fresh butter to their newborn, respectively. Close to 5% of study participants reported that they have given “Amessa”, a mixture of crushed herbs stirred with water, to their infants believing that it helps in preventing potential illnesses. Among mothers who practiced prelacteal feeding, 87 (57.2%) of them did so due to the belief that prelacteal feeds are good for newborn’s health and 45 (29.6%) did due to the belief that the first breast milk (colostrum) is not good for newborn’s health.

**Table 4:** Prelacteal feeding practice among respondents, Hawela Tula, SNNPR, 2016

Variable	Frequency (%)
Did you give any food/fluid to your newborn before the initiation of breastfeeding or in the first three days of birth?	
Yes	152 (25.5)
No	445 (74.5)
Type of prelacteal food given to newborn	
Boiled water	56 (36.8)
Fresh butter	49 (32.2)
Amessa^a^	28 (18.4)
Honey	17 (11.2)
Formula milk	2 (1.3)
Primary reason for practicing prelacteal feeding (*n* = 152)	
Perception that it is good for the newborn’s health	87 (57.2)
Perception that the first breast milk is not good for the newborn	45 (29.6)
Mother was feeling unwell	11 (7.2%)
Delayed breast milk production	5 (3.3)
Infant perceived unwell after birth	4 (2.6)

^a^a mixture of crushed herbs stirred with water

### Factors associated with prelacteal feeding

In multivarite logistic regression analysis, mothers’ knowledge on breastfeeding, knowledge on the risks of prelacteal feeding, and misconception on breastfeeding were identified to be the associated with prelacteal feeding practice (Table 5). Practice of prelacteal feeding was nine times higher among care takers who had poor knowledge on breastfeeding (AOR 8.9, 95% CI 4.2, 18.7). Additionally, care takers who lack knowledge on the risks associated with prelacteal feeding were seven times more likely to practice prelacteal feeding than their counterparts (AOR 6.8, 95% CI 2.6, 17.8). Respondents who had misconception on breastfeeding were eight times more likely to practice prelacteal feeding (AOR 8.1, 95% CI 3.9, 16.6). As to information on breastfeeding during antenatal visit, mothers who claimed no receipt of information on breastfeeding were more likely to practice prelacteal feeding (AOR 2.7, 95% CI 1.1, 6.6).

**Table 5:** Factors associated with prelacteal feeding practice, Hawela Tula, SNNPR, 2016

Variables		Prelacteal feeding practice		COR (95% CI)	AOR (95% CI)
		No	Yes		
Age of child (in months)	< 1	19 (12.5%)	42 (9.4%)	Ref.	Ref.
	2–4	74 (48.7%)	241 (54.2%)	0.7 (0.4,1.2)	0.6 (0.3,1.5)
	5–6	59 (38.8%)	162 (36.4%)	0.8 (0.4,1.5)	1.1 (0.5, 2.4)
Number of children	≤ 2	62 (40.8%)	201 (45.3%)	Ref.	Ref.
	3–4	49 (32.2%)	165 (37.2%)	0.9 (0.6, 1.4)	1.1 (0.6, 2.0)
	≥ 5	41 (27.0%)	78 (17.6%)	1.7 (1.1, 2.7)	1.2 (0.6,2.4)
Mother’s level of education	No formal education	279 (62.7%)	85 (55.9%)	Ref.	Ref.
	Some formal education	166 (37.3%)	67 (44.1%)	1.3 (0.9,1.9)	1.3 (0.8, 2.2)
Place of delivery	Health facility	301 (67.6%)	94 (61.8%)	Ref.	Ref.
	Home	144 (32.4%)	58 (38.2%)	1.3 (0.9, 1.9)	0.9 (0.6, 1.6)
Knowledgeable on risks of prelacteal feeding	Yes	345 (77.5%)	88 (57.9%)	Ref.	Ref.
	No	100 (22.5%)	64 (42.1%)	2.5 (1.7, 3.7)	6.8 (2.6, 17.8)
Ever received information on breastfeeding/infant feeding	Yes	441 (92.4%)	105 (69.1%)	Ref.	Ref.
	No	34 (7.6%)	47 (30.9%)	5.4 (3.3, 8.8)	2.7 (1.1, 6.6)
Knowledge on breastfeeding	Poor	111 (24.9%)	86 (56.6%)	12.4(6.6,23.2)	8.9 (4.2, 18.7)
	Moderate	126 (28.3%)	53 (34.9%)	6.7 (3.5,12.8)	7.8 (3.6, 16.7)
	Good	208 (46.7%)	13 (8.6%)	Ref.	Ref.
Misconception on breastfeeding	Yes	416 (93.5%)	94 (61.8%)	8.8 (5.3, 14.5)	8.1 (3.9, 16.6)
	No	29 (6.5%)	58 (38.2%)	Ref.	Ref.

AOR: Adjusted Odds Ratio, COR: Crude Odds Ratio

## Discussion

This study revealed that the prevalence of prelacteal feeding was 25.5%; boiled water (36.8%), fresh butter (32.2%) and amessa (4.7%) were the common prelacteal feeds given to newborns in the study area. Lack of knowledge on the risks associated with prelacteal feeding, poor knowledge on breastfeeding, and misconception on breastfeeding were found to be significantly associated with prelacteal feeding practice. Other Ethiopian studies in Raya Kobo district [5] revealed that butter (32.2%) and boiled water (3.7%) were the main prelacteal feeds given to newborns while plain water (22.7%) was the main prelacteal feed given to newborns in Harari region [21].

The level of prelacteal feeding in the current study (25.5%) is nearly similar to the level revealed by a study conducted in the city of Bahir Dar (27%), the capital city of Amhara Regional State [22]. This may be due to similarity of the study design deployed. Furthermore, the level of prelacteal feeding in the current study is also similar with the prevalence that was reported by the Ethiopian Demographic and Health Survey (27%) [11]. However, the level of prelacteal feeding in the current study is lower than the one conducted in Boricha district (40.1%), one of the districts in the Sidama zone, southern Ethiopia [12]. This could be due to the difference in geographical set up and access to information and healthcare; Boricha district is more remote than the current study site. The prevalence of prelacteal feeding in the current study (25.5%) is lower than the one revealed in Raya Kobo district, north eastern Ethiopia (38.8%) which might be due to the difference in study design and study participants; respondents of the Raya Kobo district were mothers of children aged less than 24 months, which may be related with recall bias which might have in turn resulted in over reporting [5].

In this study, lack of knowledge on the risks associated with prelacteal feeding was associated with prelacteal feeding practice (AOR 6.8; 95% CI 2.6, 17.8) which is in agreement with Raya Kobo study, north eastern Ethiopia where mothers who did not know the risks associated with prelacteal feeding were 3.7 times more likely to practice prelacteal feeding [4]. Similarly, findings from a study that was conducted in Mizan-Aman town, south west Ethiopia, showed that mothers who did not know the disadvantages of prelacteal feeding were about seven times more likely to practice prelacteal feeding (AOR 6.9; 95% CI 3.0, 15.9) [14].

Poor maternal knowledge on breastfeeding was associated with the practice of prelacteal feeding (AOR 8.9; 95% CI 4.2, 18.7) which is similar with the report from a study conducted in Arbaminch, south Ethiopia, where mothers who had no knowledge of breastfeeding were less likely to practice exclusive breastfeeding (AOR 0.50, 95% CI 0.03, 1.37) [23]. A Vietnamese study also showed that higher knowledge scores on breastfeeding corresponds to lower odds of feeding prelacteal foods [24]. These supporting evidences warrant that there is much to do on information, education, and communication to improve the awareness of communities on optimal infant feeding practices.

This study generated context specific evidences on practice of prelacteal feeding in the study area. However, it has some methodological limitations which need to be considered in future studies. First, since this study used a cross-sectional design, conclusion on causality couldn’t be made. Second, as mothers were asked to retrospectively recall and report their infant feeding practice in the first three days following delivery, there is some room of recall bias. Third, the self-report on infant feeding practice might have resulted in desirability bias; mothers may be less likely to report that they have practiced prelacteal feeding if they know they are not supposed to do so. Although multiple logistic regression analysis was done to control for the effect of potential confounders, there might be other potential confounding variables that were not considered in this study (residual confounders). Although our data fits well for logistic regression analysis, the association measures (odds ratios) might have been overestimated. Moreover, as this study used only quantitative approaches of assessing the level of prelacteal feeding, it is recommended if future studies consider a mixed-method design to understand the depth of the underlying factors that may be linked with the practice of prelacteal feeding.

## Conclusions

Prelacteal feeding is commonly practiced in Hawela Tula, south Ethiopia. This makes breastfeeding practices suboptimal in the area. Poor knowledge of mothers on breastfeeding, lack of knowledge on the risks of prelacteal feeding and misconception on breastfeeding are the important predictors of prelacteal feeding practice. This obviously presents a challenge to achieve the WHO recommended 90% rate of practice of exclusive breastfeeding among children under six months of age. Therefore, improving the awareness of mothers on: optimal breastfeeding, the risks associated with prelacteal feeding, and the misconceptions on breastfeeding are recommended interventions to reduce prelacteal feeding practice in the study area and other similar settings.

## Abbreviations

AOR: Adjusted Odds Ratio
CI: Confidence Interval
COR: Crude Odds Ratio
IYCF: Infant and Young Child Feeding
SNNPR: Southern Nations, Nationalities and Peoples Region
WHO: World Health Organization
